# Determination of Hydroxy Polycyclic Aromatic Hydrocarbons in Human Urine Using Automated Microextraction by Packed Sorbent and Gas Chromatography–Mass Spectrometry

**DOI:** 10.3390/ijerph192013089

**Published:** 2022-10-12

**Authors:** Samuel García-García, Héctor Matilla-González, Javier Peña, Miguel del Nogal Sánchez, Ana María Casas-Ferreira, José Luis Pérez Pavón

**Affiliations:** Departamento de Química Analítica, Nutrición y Bromatología, Facultad de Ciencias Químicas, Universidad de Salamanca, 37008 Salamanca, Spain

**Keywords:** polycyclic aromatic hydrocarbon metabolites, urine samples, microextraction by packed sorbent, gas chromatography–mass spectrometry, underivatized analytes

## Abstract

A fast methodology for the determination of monohydroxy polycyclic aromatic hydrocarbons in human urine using a fully automated microextraction by packed sorbent coupled to a gas chromatograph–mass spectrometer is reported. Sample preparation requires simple hydrolysis, centrifugation, filtration, and dilution. The method does not require a derivatization step prior to analysis with gas chromatography and allows the measurement of up to three samples per hour after hydrolysis. Quantitation is carried out by a one-point standard addition allowing the determination of 6 analytes with good limits of detection (10.1–39.6 ng L^−1^ in water and 0.5–19.4 µg L^−1^ in urine), accuracy (88–110%) and precision (2.1–23.4% in water and 5.1–19.0% in urine) values. This method has been successfully applied to the analysis of six urine samples (three from smoker and three from non-smoker subjects), finding significant differences between both types of samples. Results were similar to those found in the literature for similar samples, which proves the applicability of the methodology.

## 1. Introduction

Polycyclic aromatic hydrocarbons (PAHs) are a group of organic pollutants formed by two or more benzene rings fused together. They can be found widespread in the environment, and they are known to produce mutagenic, carcinogenic, or teratogenic effects [1,2]. PAHs can be released into the environment from both natural and anthropogenic sources such as incomplete combustion of fossil fuels, natural gas, wood, coke, and organic waste, and some studies have shown that high concentrations of these compounds can also be found in grilled meat [3]. Once PAHs enter the human body, either by ingestion, dermal contact, or inhalation, they are rapidly metabolized into their corresponding hydroxylated derivatives (OH-PAHs) and conjugated to a sulfate or glucuronide form, which will be excreted via urine or feces, making OH-PAHs good biomarkers of PAHs [4,5]. These OH-PAHs can be even more harmful to human health than their corresponding parent PAHs [6], and they may be bio-transformed to reactive electrophiles, which can bind to DNA [7]. These metabolites have relatively short half-life elimination times (5–35 h), and they are useful for assessing recent exposure to PAHs [8]. On many occasions, a single metabolite, usually 1-hydroxipyrene, has been used to assess PAH exposure levels. However, this strategy cannot fully represent comprehensive exposure levels to PAHs since, in a real situation, the number of analytes is higher. The determination of a wide variety of metabolites has advantages in exposome studies [9].

Gas chromatography (GC) [8,10,11,12] and liquid chromatography (LC) [13,14,15] coupled with mass spectrometry (MS) have been commonly used for the determination of OH-PAHs in urine samples. However, after the corresponding hydrolysis to liberate the OH-PAHs from their conjugate forms [16], typical sample preparation in gas chromatography-based methods requires a derivatization step using organic reagents since OH-PAHs are not volatile enough due to their polarity and molecular weight, making the developed methods so far labor-intensive, more expensive, and less environmentally friendly, compared to the strategy we propose here. To the best of our knowledge, to date, there is only one article with a method based on GC-MS where there is no derivatization of OH-PAHs before chromatographic analysis [10], where solid-phase microextraction (SPME) is used to extract the analytes, which requires more than 45 min per sample. The method we propose takes only 17.5 min per sample, including not only the extraction step but also the chromatographic analysis. Regarding analyte extraction, attention has also been paid to improving sample treatment techniques to separate compounds from the sample matrix and enrich them effectively. In this sense, several solid-phase microextraction techniques have been used for the enrichment of OH-PAHs [10,14,17]. An alternative that has not been extensively explored to date in this field is the use of microextraction by packed sorbent (MEPS), which is based on the miniaturization of conventional solid-phase extraction [18]. Another option to improve sensitivity is the use of a programmed temperature vaporizer (PTV) [18,19] to inject the samples into the gas chromatograph. In this case, the metabolites are retained in the liner by cold trapping, while other matrix components are eliminated through the split valve using the solvent vent mode. Some authors [20] have eliminated the step of chromatographic separation of the metabolites, and the whole sample is introduced directly into the mass spectrometer. In this case, the method was based on SPME coupled to glass-capillary nanoelectrospray ionization mass spectrometry (nanoESI-MS).

In this work, a rapid and sensitive method for the determination of underivatized monohydroxy polycyclic aromatic hydrocarbons in human urine samples is reported. The method is based on a fully automated microextraction by packed sorbent coupled to gas chromatography (including programmable temperature vaporization) and mass spectrometry with no derivatization step, which simplifies laboratory work and reduces analysis time and cost, and it is more environmentally friendly since no organic reagents are used. In addition, this reduces the error related to the sample preparation step prior to chromatographic analysis due to the minimal sample manipulation.

## 2. Materials and Methods

### 2.1. Reagents and Standards

Standards of 1-hydroxynaphthalene (1-NAP, C_10_H_8_O, 99%), 2-hydroxynaphthalene (2-NAP, C_10_H_8_O, 99%), 1-hydroxyacenaphthene (1-ACE, C_12_H_10_O, 99%), 2-hydroxyfluorene (2-FLU, C_13_H_10_O, 98%), 9-hydroxyfluorene (9-FLU, C_13_H_10_O, 96%), 9-hydroxyphenanthrene (9-PHE, C_14_H_10_O, tech. grade), enzyme β-Glucuronidase from Helix pomatia (Type H-5, lyophilized powder, ≥400,000 units/g solid), methanol (HPLC grade, 99.9%) and ethyl acetate (HPLC grade, 99.9%) were supplied by Sigma-Aldrich (Steinheim, Germany). For this study, 1-Naphthol β-d-glucuronide sodium salt (C_16_H_15_NaO_7_, 98%) was obtained from Toronto Research Chemicals Inc.

Stock solutions (1000 mg L^−1^) of each standard were prepared in methanol and stored at −20 °C prior to use. Dilution to working standard concentrations was made using ultra-high-quality water (UHQ, obtained with a Wasserlab Ultramatic water purification system, Noain, Spain). All solutions were stored at 8 °C. Before use, these solutions were left to warm up to room temperature and subsequently diluted to prepare the working solutions for spiking samples.

### 2.2. Synthetic Urine and Urine Samples

Synthetic urine was prepared following the procedure described by van de Merbel [21]. Human urine samples were obtained from six subjects (three non-smoker and three smoker), in a disposable sterile collection cup and stored at −20 °C until analysis (typically 24–72 h). All subjects were told to collect the first sample in the morning. After thawing at room temperature, samples were vortexed at maximum speed for 1 minute for homogenization prior to use. Informed consent was obtained from all subjects involved in the study prior to analysis.

### 2.3. Hydrolysis, MEPS Extraction Procedure, and Instrumental Conditions

For analysis, urine samples were thawed at room temperature and transferred to a 12-mL polypropylene tube (Scharlab), followed by vortexing for 1 min at room temperature. From the mixture, 800 µL were transferred to a 10 mL headspace vial. Here, 100 µL of enzyme solution (5000 uds/mL in pH 5 buffer) and 100 µL of UHQ water were added. The vial sample was introduced in an oven at 37 °C for 15 hours for hydrolysis. Next, the sample was again transferred to a 12-mL polypropylene tube and centrifuged for 5 min at 4500 rpm (1815 g). The supernatant was filtered using a polytetrafluoroethylene filter (PTFE, 0.45 µm, 17 mm i.d.), and 500 µL of the filtrate were added to a new 10 mL headspace vial, followed by 2000 µL of a pH 7 buffer. The mixture was then subjected to the MEPS extraction procedure.

An MPS2 Multi-Purpose Sampler (Gerstel, Mülheim an der Ruhr, Germany) was used to perform all the next steps automatically from here. Extraction of the analytes was performed using a C18 cartridge (SGE Analytical Science). Optimal conditions for MEPS were found to be as follows: conditioning of the sorbent with 100 µL of ethyl acetate; then, passing 100 µL of UHQ water (flow rate, 25 µL/s). Next came extracting the sample (3 cycles of 100 µL at a flow rate of 5 µL/s using the drawing and discarding mode). The next step was to then wash the sorbent with 100 µL of UHQ water to remove interferences and drying the cartridge by pumping air through it (10 × 100 µL) at a flow rate of 25 µL/s. The compounds were eluted with 30 µL of ethyl acetate and injected into the programmed temperature vaporizer (PTV) filled with an empty deactivated baffled glass liner (71 mm × 2 mm I.D., Gerstel CIS-4) at a flow rate of 5 µL/s.

Solvent vent mode was selected for inlet operation using the following conditions: initial temperature of 80 °C for 0.55 min with a vent flow of 50 mL min^−1^ (2.00 psi). After venting the solvent, the split valve was closed, and the liner was flash-heated (12 °C/s) up to 270 °C to transfer the analytes into the column. Injection time was set to 2.0 min. The temperature was kept for 5 min after injection with the split valve opened for cleaning, using a split vent purge flow of 50 mL min^−1^. Finally, liquid CO_2_ (Air Liquide) was used to reach the initial conditions again.

**Figure 1:**
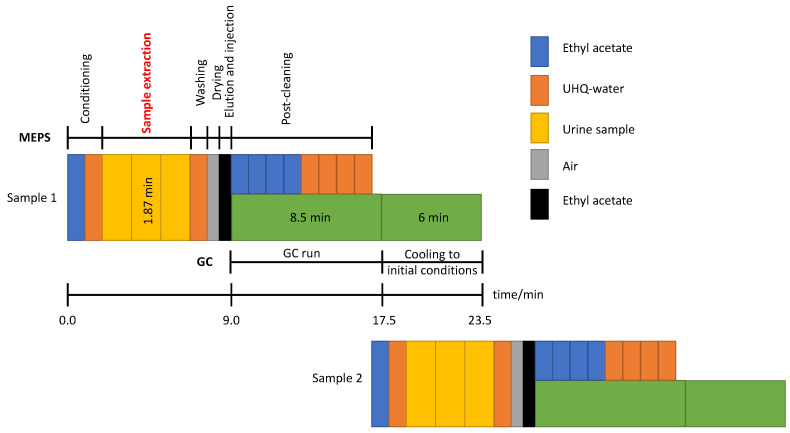
Scheme of the MEPS steps for two consecutive samples.

Analyses of OH-PAHs were performed on a GC-MS instrument (Agilent Technologies, Santa Clara, CA, USA) consisting of a 6890 gas chromatograph interfaced with an HP 5973 N quadrupole mass spectrometer. Chromatographic separation was carried out using an HP-5MS UI capillary column (30 m × 0.25 mm, 0.25 µm; J&W Scientific, Folsom, CA, USA). A flow rate of 1 mL min^−1^ of Helium N50 (99.9999 % pure, Air Liquid) as the carrier gas was used. The initial oven temperature was set at 80 °C and was held for 1 min. Then, the temperature was increased from 30 °C min^−1^ to 300 °C and held for 1.5 min. The total chromatographic run time was 8.5 min. The total time needed for analysis per sample was 17.5 min: 9 min for the entire MEPS process (excluding reconditioning since this is automatically overlapped during the GC run; see Figure 1) and 8.5 min for chromatographic separation.

The MS detector was equipped with an inert ion source and operated in electron ionization mode using an ionization voltage of 70 eV. The ion source temperature was 230 °C, the quadrupole was set at 150 °C, and the transfer line temperature was 280 °C. The analyses were performed in a synchronous SIM/Scan mode, acquiring both SIM and Full Scan data in a single run (solvent delay 4.00 min). Full Scan (*m*/*z* range: 60–250 amu) was used for identification and SIM for quantification, selecting the characteristic ions in each case (one quantification and two qualifier ions were monitored), with a dwell time of 10 ms. MSD ChemStation, Ver. E.02.00.493 software from Agilent Technologies was used for data acquisition. NIST_98 (NIST/EPA/NIH Mass Spectral Library, version 2.0) database was used for identification.

## 3. Results and Discussion

Several factors affecting sample preparation, MEPS procedure and PTV-GC-MS analysis were optimized. For the optimization studies, unless otherwise stated, the instrumental conditions used were the ones in Section 2.3.

### 3.1. Optimization of Sample Preparation—Matrix Effect

In order to determine OH-PAHs in urine samples, these must be firstly hydrolyzed to deconjugate the corresponding glucuronide and sulfate conjugates. A procedure similar to the one described by Li et al. was followed [16]. For optimization studies, 800 µL of a urine sample spiked with 50 µL of 1-naphthol β-d-glucuronide (5 mg L^−1^) was used and diluted to a final volume of 1 mL with different volumes of a β-glucuronidase solution (5000 units mL^−1^). This way, the corresponding matrix effect from urine is still being taken into account and, due to the considerable amount of 1-NAP conjugate used for spiking, the amount of this analyte already present in the urine sample naturally would not affect the results observed.

After optimization, 0.5 units per microliter of the sample was chosen as the optimal amount of enzyme to be used. The hydrolysis was tested at different times at 37 °C, finding that after 8 hours, the process was completed, and no significant difference with 15 hours (overnight) was observed. However, we decided to keep the hydrolysis overnight to have a more reliable procedure and to ensure that all OH-PAHs conjugates are completely hydrolyzed.

Regarding a centrifugation step, no significant difference was observed between a non-spiked urine sample submitted to a centrifugation step (4500 rpm for 5 min) or not submitted, indicating that most of the analytes are present in the supernatant fraction of the sample. We decided to keep the centrifugation step to remove most of the solid particles of the urine and help with the following steps: filtration and extraction. In addition, solutions with different final pH values were tested since the MEPS cartridge best works between pH 2 and 8. Hence, pH values of 5, 6, 7, and 8 were evaluated in two different urine samples spiked with 1 µg L^−1^ of OH-PAHs. Depending on the urine sample, similar results are obtained for most of the analytes, but for the most polar ones such as 1-NAP or 2-NAP, smaller signals appear as the pH increases. As a compromise value, pH 7 was selected, where the best signal was obtained for all the analytes.

**Figure 2:**
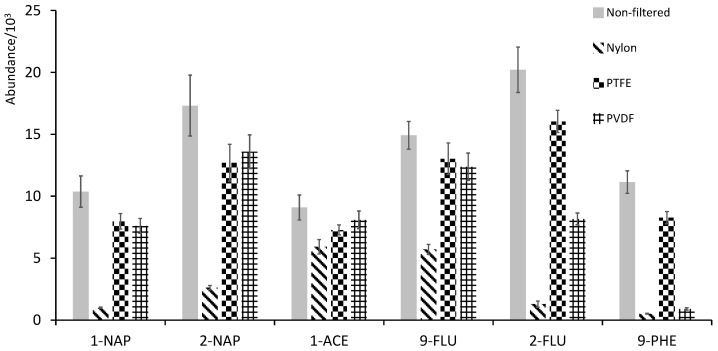
Test of filter materials.

For filtration, Nylon, polytetrafluoroethylene (PTFE), and polyvinylidene fluoride (PVDF) were tested as filter materials (0.45 µm) using urine spiked with 1 µg L^−1^ of OH-PAHs. Figure 2 shows a comparison of different filter materials against a non-filtered urine sample, and, as shown, Nylon retains most of the analytes. However, PTFE and PVDF behaved similarly with more polar analytes, although PVDF greatly retained the least polar. Hence, PTFE was chosen as the filter material.

Dilution ratios of 1:2, 1:5, and 1:10 were evaluated, but none of these were able to eliminate the matrix effect. However, considering the following MEPS step, a 1:5 dilution factor was kept for urine preparation to protect the cartridge. The matrix effect was evaluated with a two-level standard addition calibration using a urine sample treated as described in Section 2.3 (that is, including filtration and dilution) and compared to a UHQ-water sample and synthetic urine [21] spiked with the same concentration level and following the same standard procedure. Table 1 shows a comparison between the slopes of the calibrations carried out in the different matrices (in this table, data for urine diluted 1:5 are shown, but similar results are obtained with dilution ratios of 1:2 or 1:10). As shown, there is a significant difference among the different matrices, proving the existence of matrix effect. Hence, a one-level standard addition method was proposed, which has been previously proven to be successful in eliminating the matrix effect [22,23]. 

### 3.2. Optimization of the MEPS Procedure

For the microextraction process, loading, washing, and elution conditions were optimized as well as different dilution ratios since this can considerably affect the extraction due to the nature of the urine sample. For loading, we tested a combination of different dilution factors (1:10, 1:5, 1:2) with different numbers of extraction cycles, from 1 × 100 µL (100 µL total sampling volume) to 10 × 100 µL (1000 µL total sampling volume), using a urine sample spiked with 10 µg L^−1^ of OH-PAHs. It should be noted that it is expected to find different concentration levels for each analyte in the urine samples. MEPS cartridge was conditioned with 100 µL of EtOAc and 100 µL of UHQ-water using the drawing and discarding mode to load the sample. As expected, as the dilution ratio increases for a fixed number of extraction cycles, the signal for all analytes decreases. Similarly, for a fixed dilution ratio, a better signal is obtained with more extraction cycles. In considering the time needed for every extraction step and reproducibility, optimal conditions were chosen as 1:5 dilution ratio and three extraction cycles. In addition, the sampling flow rate was evaluated, and 5 µL s^−1^ was selected as optimum.

**Figure 3:**
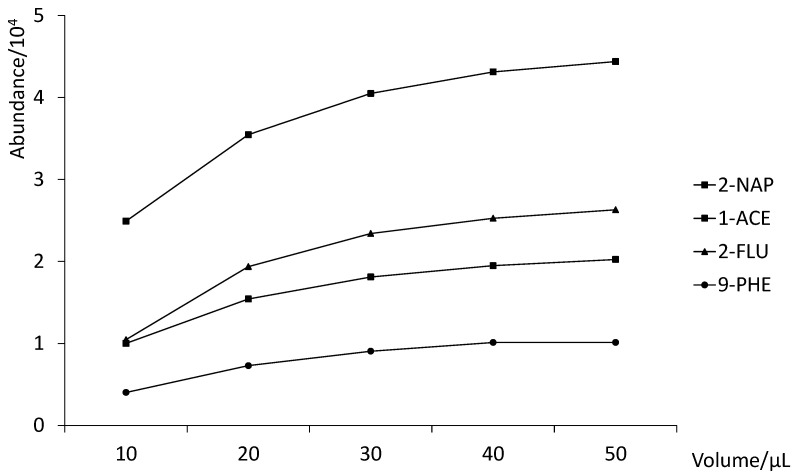
Optimization of MEPS elution solvent volume.

In order to remove all plausible interferences, a washing step is of remarkable importance. This was evaluated using a synthetic urine spiked with 1 µg L^−1^ of the analytes, passing different 100 µL mixtures of UHQ-water and methanol, 100:0, 90:10, 80:20, and 70:30 through the C18 cartridge. The best results were obtained with pure UHQ-water since the presence of methanol decreased the signal for most of the OH-PAHs due to higher elution force of the organic solvent. For elution, we decided to use the same solvent used previously for the determination of PAHs [18]. Hence, different volumes of ethyl acetate (10, 20, 30, 40 and 50 µL) were evaluated with an elution flow rate of 5 µL/s. As shown in Figure 3 (1-NAP and 9-FLU are not shown because their results overlap with 2-NAP and 2-FLU), 30 µL is enough to elute more than 90% of the analytes from the C18 sorbent (100% of elution was considered for 50 µL of solvent, where no significant increase of signal was observed, compared to a smaller elution volume tested).

When using MEPS for preconcentration, a common problem is typically carry-over. This was evaluated by washing four times with 100 µL of EtOAc and four times with 100 µL of UHQ-water after the elution of compounds from the C18 cartridge. In comparing chromatograms of a urine sample spiked with 1 µg L^−1^ of each analyte and a chromatogram from the next elution just after washing, no carry-over was detected for any of the analytes. With the conditions described, the MEPS cartridge could be reused for around 80 extractions.

### 3.3. PTV-GC-MS Conditions

Due to the use of MEPS for preconcentration, solvent vent injection mode was selected to remove the excess organic solvent used for elution. Vent time and flow, injection time, and PTV initial temperature (vent temperature) were the factors studied. Vent time was evaluated from 0.5 to 1.0 min, vent flow from 50 to 100 mL min^−1^, injection time from 2.0 to 3.0 min, PTV initial temperature from 70 to 100 °C, keeping PTV final temperature (injection temperature) at the maximum recommended by the liner manufacturer, that is, 270 °C. Optimal conditions, in terms of better signal and reproducibility, were found to be the following: vent time 0.50 min; vent flow 50 mL min^−1^; injection time 2.0 min; PTV initial temperature 80 °C.

Regarding gas chromatography, previous conditions used for the determination of PAHs were taken as a starting point for optimization [19]. From here, different initial temperatures (80–100 °C) and times (1.00–1.20 min) were evaluated, keeping the final temperature at 300 °C, in order to avoid column degradation since the maximum temperature recommended by the manufacturer is 325 °C. Temperature ramps used were always the maximum permitted by the system. Best results were obtained at a lower temperature, and no significant difference was observed with increasing time, so 80 °C and 1 min were selected.

For MS analysis, SIM/Scan synchronous mode was selected, evaluating different sampling rates (N = 0, 1, 2, and 4) and dwell time values (1, 10, 50, and 100 ms). The best morphology was observed with a value of N = 2. Regarding dwell times, 10 ms was chosen as optimum since it gave the best signal/noise ratio. Table 2 shows the SIM groups with the ions selected for quantitation and identification of all the analytes.

### 3.4. Method Validation

The proposed method was evaluated in terms of limits of detection (LOD), limits of quantitation (LOQ), linearity, repeatability (intra- and inter-day precision) and accuracy. The study was performed both on UHQ-water and urine samples.

For calibration in UHQ-water, six concentration levels were measured in triplicate, using extracted quantitation ions from SIM chromatograms to integrate areas of each analyte. For urine, a two-point standard addition method was used. As shown in Table 3 and Table 4, good linear behavior (R^2^ values > 0.99) was obtained. None of the calibrations was a lack of fit. Since the analytical signals were affected by the nature of the sample, both LODs and LOQs values were matrix dependent. Furthermore, none of the samples was fully free from all of the analytes; thus, LODs and LOQs were calculated for the different urine samples analyzed. Signal to noise ratio (S/N = 3 for LOD and S/N = 10 for LOQ) was used following ISO 11843-1 [24]. Table 3 and Table 4 show good LODs and LOQs values both in UHQ-water and urine (expressed as concentration range found in the different samples analyzed), in the ng L^−1^ or low µg L^−1^ range, respectively. Intra- and inter-day repeatability studies were done with UHQ-water and urine samples, both equally spiked at low (5–25 µg L^−1^) and high (30–90 µg L^−1^) concentrations levels. Intra-day repeatability was assessed by measuring one UHQ-water sample and two urine samples on the same day, four times each, while inter-day repeatability was measured by analyzing one UHQ-water sample and two urine samples, prepared each day, during 2 consecutive days, four times each. As shown, the results expressed as mean %RSD ranged from 2.1% to 23.4% in UHQ-water and from 5.1 to 19.0% in urine, which can be acceptable considering the reproducibility of the MEPS procedure. The use of internal standard did not improve the results in terms of precision. Thus, we decided not to include it in order to have a simpler sample preparation procedure. With these conditions, intra-day repeatability RSD values were kept below 10%. For recovery assessment, one UHQ-water and two urine samples were spiked at two different levels (low: 5–25 µg L^−1^; high: 30–90 µg L^−1^), and they were submitted to the standard procedure. Table 5 shows the recoveries for each analyte as percentage values calculated by comparing the areas of each chromatogram to the corresponding ones from ethyl acetate solutions spiked with the same concentrations. Good results are obtained both in UHQ-water and urine matrices. 

The accuracy of the method was determined as the ratio of the found concentration to the added concentration (apparent recovery, as a percentage) in four spiked urine samples, subtracting the signals from the non-spiked, hydrolyzed samples in every case. Acceptable mean values were found between 88 and 110% (Table 6), proving the applicability to determine OH-PAHs in urine using the proposed methodology.

### 3.5. Analysis of Urine Samples

**Figure 4:**
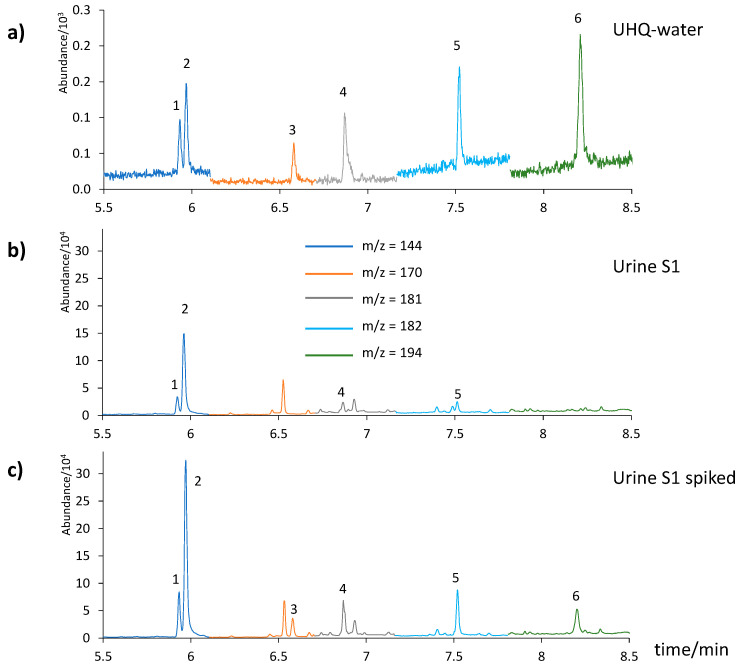
Chromatograms of UHQ-water spiked at the LOQ level in water (**a**), compared to a urine sample analyzed (S1), non-spiked (**b**) and spiked (**c**). 1= 1-NAP, 2 = 2-NAP, 3 = 1-ACE, 4 = 9-FLU, 5 = 2-FLU, 6 = 9-PHE.

To prove the viability of our proposed method, we applied it to the analysis of six urine samples (three non-smoker and three smoker). As shown in Table 7, there is a significant difference between the smoker (S1, S2, and S3) and non-smoker subjects (S4, S5, and S6), especially for 1-NAP and 2-NAP. Only 1-NAP, 2-NAP, 2-FLU, and 9-FLU were found in the urine sample analyzed, in concentrations which were in agreement with those reported in the literature [25,26,27,28]. The other OH-PAHs were not found. Figure 4 shows the chromatograms corresponding to UHQ-water at the LOQ level in water (Figure 4a) and urine sample S1, both non-spiked (Figure 4b) and spiked (Figure 4c). Only 1-ACE and 9-PHE were not detected naturally in these urine samples. In relation to the quantitation of 2-FLU, there is an interference next to it due to a natural compound present in most of the urine samples analyzed. In any case, the maximum calculated error when integrating the corresponding area would be 15%. It is noteworthy the correlation between the values previously observed by our group and others for PAHs in saliva and the corresponding metabolites in urine. In this sense, naphthalene and fluorene derivatives were observed again as major components, and acenaphthylene derivative was not detected [29,30].

### 3.6. Comparison with Other Works

Most methods for the determination of OH-PAHs in urine require a derivatization step prior to analysis with gas chromatography, and only two publications can be found in the literature where this step is avoided [10,20]. In one case, extraction is performed by immersing an SPME fiber in the sample for 45 min plus 3 min more for desorption, with a GC run lasting more than 17 min. Our procedure is short and only takes 9 min for the MEPS procedure (initial conditioning with EtOAc and UHQ-water, 3 extractions, washing off interferents and drying). Thanks to the automatic robot used, it allows performing reconditioning of the cartridge and loading of the next sample while the chromatographic separation is run, saving extra time and allowing to measure three samples per hour, being the fastest method described yet, with no derivatization. The chromatographic run time only takes 8.5 min and allows the determination of up to six different analytes, including isomers such as 1-NAP and 2-NAP or 2-FLU and 9-FLU. In the second literature reference, SPME is also used to extract the analytes, which are introduced into a triple quadrupole/linear ion trap mass spectrometer using electrospray ionization. This work reports the determination of five OH-PAHs, but no structural isomers can be determined since there is no chromatographic separation.

Comparing our method to those based on LC-MS/MS, which are the most sensitive methods found in the present literature [13,17,31], our performance in urine is similar in terms of accuracy, precision, and chromatographic runtime. Although our LODs are not as low, they are still in the range of the ng mL^−1^, except for 9-PHE. Furthermore, many of the LC-MS/MS methods have a manual sample preparation, which is time consuming. Compared to these, our method is fully automated, making the sample preparation easier and faster.

## 4. Conclusions

In this work, we present the first method for the determination of 6 hydroxy metabolites of polycyclic aromatic hydrocarbons in urine without derivatization, using a fully automated MEPS procedure coupled with a gas chromatograph-mass spectrometer. This methodology presents good LODs and LOQs, accuracy and precision and allows the analysis of several samples per hour, proving to be the fastest method to determine these analytes in urine compared to other works, to the best of our knowledge. In order to prove its validity, it has been applied to six urine samples from both smokers and non-smokers, showing a significant difference between these subjects, especially for the concentration of naphthalene derivatives. The results are in agreement with those reported previously by our and other research groups and prove the applicability of the proposed methodology.

## Figures and Tables

**Table 1:** Comparison of the slopes found for the linear equations obtained with UHQ-water, synthetic urine, and three urine samples from subjects S1, S2, and S6.

Analyte	UHQ-Water	Synthetic Urine	S1	S2	S6
1-NAP	42.0 ± 0.8	35 ± 1	58 ± 2	50 ± 2	27 ± 8
2-NAP	75 ± 2	66 ± 3	111 ± 6	106 ± 3	143 ± 9
1-ACE	48 ± 2	42 ± 2	67 ± 3	73 ± 2	57 ± 6
9-FLU	57 ± 2	48 ± 2	94 ± 3	104 ± 4	124 ± 5
2-FLU	72 ± 2	63 ± 3	94 ± 7	98 ± 5	102 ± 2
9-PHE	44 ± 2	27 ± 1	16 ± 2	14 ± 5	19 ± 3

For concentration ranges in each matrix please, see Section 3.4.

**Table 2:** Retention times, selected ions for quantification (in bold) and identification of OH-PAHs including internal standard, and SIM groups used.

Compound	tR/min	Quantifier and Qualifier Ions	SIM Group
1-NAP	5.89	**144**, 115, 116	1
2-NAP	5.93	**144**, 115, 116	
1-ACE	6.56	**170**, 169, 152	2
9-FLU	6.85	**181**, 182, 152	3
2-FLU	7.48	**182**, 181, 152	
9-PHE	8.16	**194**, 165, 166	4

**Table 3:** Analytical characteristics of the proposed method for OH-PAHs in UHQ-water.

Compound	Calibration Range (ng L^−1^)	Linear Equation	R^2^	LOD (ng L^−1^)	LOQ (ng L^−1^)	Intra-Day Repeatability (RSD, %)		Inter-Day Repeatability (RSD, %)	
						Low Level ^a^	High Level ^b^	Low Level ^a^	High Level ^b^
1-NAP	LOQ-800	y = (42 ± 1) x + (2 ± 3) 10^2^	0.9985	14.9	49.7	9.7	4.9	23.4	8.3
2-NAP	LOQ-500	y = (75 ± 2) x + (0 ± 6) 10^2^	0.9956	11.6	38.7	7.1	4.3	13.3	4.9
1-ACE	LOQ-1200	y = (48 ± 2) x − (0 ± 1) 10^3^	0.9930	14.6	48.6	5.7	2.7	13.0	3.3
9-FLU	LOQ-1200	y = (57 ± 2) x + (0 ± 1) 10^3^	0.9957	10.1	33.6	5.9	2.1	11.9	6.0
2-FLU	LOQ-1200	y = (72 ± 2) x − (0 ± 1) 10^3^	0.9954	10.8	36.0	9.0	4.5	12.0	11.5
9-PHE	LOQ-18000	y = (44 ± 2) x − (0 ± 1) 10^4^	0.9932	39.6	132.2	9.2	5.7	10.8	14.3

^a^ Concentrations in level LOW: 1-NAP, 2-NAP, 1-ACE, 9-FLU, 2-FLU: 5 µg L^−1^; 9-PHE: 25 µg L^−1^; ^b^ Concentrations in level HIGH: 1-NAP, 2-NAP, 1-ACE, 9-FLU, 2-FLU: 60 µg L^−1^; 9-PHE: 90 µg L^−1^.

**Table 4:** Analytical characteristics of the proposed method for OH-PAHs in urine.

Compound	Calibration Range (µg L^−1^)	R^2^	LOD * (µg L^−1^)	LOQ * (µg L^−1^)	Intra-Day Repeatability (RSD, %)		Inter-Day Repeatability (RSD, %)	
					Low Level ^a^	High Level ^b^	Low Level ^a^	High Level ^b^
1-NAP	LOQ-62	0.9977	0.6–4.4	2.2–14.7	7.0	8.5	12.7	7.6
2-NAP	LOQ-375	0.9989	0.7–2.6	2.3–8.5	6.3	4.8	11.1	9.4
1-ACE	LOQ-62	0.9947	0.7–7.3	2.2–24.0	6.0	5.1	10.6	9.6
9-FLU	LOQ-75	0.9955	0.7–5.4	2.2–18.3	5.8	3.7	11.8	10.3
2-FLU	LOQ-60	0.9990	0.5–2.1	1.5–7.0	5.7	6.3	19.0	14.9
9-PHE	LOQ-125	0.9939	6.1–19.4	20.6–65.6	7.9	6.5	14.7	8.9

* LOD and LOQ values expressed as concentration range found in the different samples analyzed; ^a^ Concentrations in level LOW: 1-NAP, 2-NAP, 1-ACE, 9-FLU, 2-FLU: 5 µg L^−1^; 9-PHE: 25 µg L^−1^; ^b^ Concentrations in level HIGH: 1-NAP, 2-NAP, 1-ACE, 9-FLU, 2-FLU: 60 µg L^−1^; 9-PHE: 90 µg L^−1^.

**Table 5:** Mean recovery values (%) obtained in UHQ-water and two different spiked urine samples (S4 and S6).

Compound	UHQ-Water		S4		S6	
	Low Level ^a^	High Level ^b^	Low Level ^a^	High Level ^b^	Low Level ^a^	High Level ^b^
1-NAP	97	89	79	78	68	64
2-NAP	78	74	52	60	49	52
1-ACE	52	59	43	52	36	41
9-FLU	66	64	57	63	51	53
2-FLU	68	81	57	75	51	62
9-PHE	48	70	15	17	11	15

^a^ Concentrations in level LOW: 1-NAP, 2-NAP, 1-ACE, 9-FLU, 2-FLU: 5 µg L^−1^; 9-PHE: 25 µg L^−1^; ^b^ Concentrations in level HIGH: 1-NAP, 2-NAP, 1-ACE, 9-FLU, 2-FLU: 60 µg L^−1^; 9-PHE: 90 µg L^−1^.

**Table 6:** Mean accuracy values (%) obtained for four different spiked urine samples.

Compound	Added Concentration (µg L^−1^)	Found Concentration (µg L^−1^)	Accuracy (%)
1-NAP	3.2	3.1 ± 0.2	97
2-NAP	9.9	9 ± 1	91
1-ACE	3.2	2.8 ± 0.2	88
9-FLU	2	1.8 ± 0.1	90
2-FLU	2.1	2.1 ± 0.1	100
9-PHE	10.0	11 ± 3	110

**Table 7:** Found concentrations in µg L^−1^ of four of the six OH-PAHs in six urine samples collected from exposed (S1, S2, and S3) and non-exposed volunteers (S4, S5, and S6). 1-ACE and 9-PHE were found below of detection limits of the method in all samples.

Subject	1-NAP	2-NAP	9-FLU	2-FLU
S1	17.7 ± 0.9	56 ± 5	3.8 ± 0.7	4.2 ± 0.5
S2	6 ± 2	37 ± 6	ND	4.3 ± 0.4
S3	13 ± 3	52 ± 9	7 ± 2	3 ± 2
S4	7 ± 1	9 ± 2	ND	<LOQ
S5	<LOQ	14 ± 3	3.2 ± 0.5	ND
S6	ND	20 ± 3	ND	ND

ND = Not detected.

## Data Availability

The data presented in this study are available on request from the corresponding author.
